# Developmental Strategies of the Promotion Policies in Medical Tourism Industry in South Korea: A 10-Year Study (2009–2018)

**Published:** 2019-09

**Authors:** Kyoung-Lee KIM, Byung-Ro SEO

**Affiliations:** 1. Asia Contents Institute, Konkuk University, Seoul, Korea; 2. Korea Department of Global MICE, Konkuk University, Seoul, Korea

**Keywords:** Medical tourism, Medical tourism industry, Medical service

## Abstract

**Background:**

After the revision of the Medical Service Act, Article 27-2, in 2009, Korea has been actively involved in the medical tourism industry. The number of foreign patients visiting Korea has consistently increased coming from various countries around the world. Currently, the industry is striving to achieve qualitative growth in medical tourism. Accordingly, there is a necessity to analyze and review policies for the development of medical tourism to create a sustainable market.

**Methods:**

We analyzed the information of foreign patients visiting Korea over a period of last 10 years. Statistical data were obtained from the Korea Health Industry Development Institute (KHIDI) and Korea Tourism Organization (KTO).

**Results:**

The number of foreign patients visiting Korea had increased from 60,201 in 2009 to 321,574 in 2017. Since 2017, the number of patients from the U.S., Russia and Middle East has been on an upward trend while the number of Chinese patients has decreased. This result clearly shows that international affairs and cultural aspects have a significant impact on the selection of medical tourism.

**Conclusion:**

It is suggested to i) establish the medical tourism information system and brand the Korean medical tourism offering a joint treatment of western and oriental medicine, ii) prepare the expansion of the industry to the medical wellness tourism industry by training global healthcare experts and iii) organize tailored medical services with consideration of various backgrounds and culture of foreign patients.

## Introduction

While the world has rapidly adopted the paradigm of the fourth industrial revolution, the secret of eternal life, the most intrinsic desire of human, is still not clear. The desire to extend life has emerged in different areas of health care including cancer surgery and internal organ transplantation. Accordingly, patients suffering from disease and aging invest on better quality of medical services, traveling around the world. The 21^st^ century medical tourism industry promoting country-specific medical service is sustained by the seamlessly interconnected medical care worldwide.

The medical tourism industry connecting cutting-edge medical services with tourism creates massive economic value in the process of consultation, entrance, therapy, relaxation, and cultural consumption, and promotes tourism and trade. It was expected that the Asian market for medical tourism would increase 20% annually and account for a $4.4 billion market by 2012 (1). Currently, it is estimated that the Asian market will record more than $14 billion in revenues by 2022 (2). The revenue of the world medical tourism market was approximately $10 billion in 2012, which was 2.5-fold higher than that in 2004, and is expected to be around $33 billion in 2019. Southeast Asia is one of the popular regions for medical tourism. The medical tourism destinations mostly provide high-quality medical services at a low cost and have short waiting time, and medicine is combined with the relaxation services (3). However, recent participants have shown big progress since 2014. Japan launched the Medical Excellence together with China to attract Middle Eastern patients. Taiwan actively attracts tourists from mainland China without any linguistic or cultural barriers. Korea recorded significant progress and considered 2014 as a year of quantum leap to attract one million foreign patients ushering in “the new era of three states in the Asian medical tourism market” (4).

Several countries adopt a country-level strategy for the industry to enhance national reputation and increase the economic value through medical services. According to the Korea Health Industry Development Institute (KHIDI) (2017), the number of foreign patients visiting Korea had increased from 27,400 in 2008 to 159,464 in 2012 and 364,189 in 2016. The increase results from medical tourism policies, established by the government and affiliated organizations, to actively cooperate with medical facilities. According to Laszlo Puczko, director of strategy department at Resources for Leisure Assets, a global medical consulting agency, “Korea already has good medical technology; however, to attract overseas patients, it needs to amend policies.” At the Medical Korea 2019 event held in March 2019, he referred to the increased cooperation between hospitals and the government to transform the Korean medical tourism sector to provide world class services (5). Accordingly, this study analyzed the current policies and the status of medical tourism industry in the recent 10 years.

## Methods

Statistical data were obtained from the Korea Health Industry Development Institute (KHIDI) and Korea Tourism Organization (KTO).

## Results and Discussion

### Medical Tourism Policy in East Asia Policy for Medical Tourism Industry in Southeast Asia

The medical tourism industry has yielded successful results in East Asia because each country in East Asia consolidated its policies for the medical tourism industry in accordance with the medical facilities and services proposed, and tourism resources available. Two countries, Thailand and Singapore, showed outstanding results. Thailand offers abundant resources for tourism; however, the efficiency of the public health care system is relatively low. The government, therefore, adopted a policy to attract domestic and foreign investment for the establishment of a high quality of medical infrastructure (6). Singapore maintains its medical competitiveness at the national level by allowing for-profit hospitals to offer high-quality medical services beyond the medical safety net through the Singapore Medicine Plan campaign (7). In China, factors such as high cost, poor infrastructure, inadequate policies and regulations, defective advertising, lack of professional skills, limited investment potential, and communication issues are still a hindrance to the development of medical industry (8). Japan is considered as a successful latecomer as its Ministry of Economy, Trade and Industry launched the Service Tourism Research Association with the New Development Strategy in 2009 and attracted the wealthy patients from China and Russia. This trend is strongly apparent from the total number of visas for medical stay (1,383) issued by the country in 2017 (9, 10). The medical tourism industry in Asia has become a competitive field. Nonetheless, it seems that Korea, offering cutting-edge medical equipment and services, has been among the top destinations for medical tourism (Korea was awarded as the destination of the year in the IMTJ Medical Travel Awards 2018).

### Policy for Medical Tourism in Korea

According to the International India Medical Tourism Congress (2013), the medical tourism industry would have grown at an annual average rate of 9.9% from 2013 to 2017 and account for 16% of the total tourism revenue in 2017. It is not a coincidence that more than 50 countries promote the medical tourism industry at the governmental level (11). The medical tourism industry in Korea began with the Vision and Development Strategy of Health Industry formulated by the Ministry of Health and Welfare in 2000 to enter the top 10 developed countries offering medical tourism. In 2007, the Korea International Medical Association was launched with 30 medical facilities at the general hospital level; however, it was hard to attract patients continuously. In 2009, the Korean government selected health care as one of the 17 new growth engines by the Ministry of Health and Welfare and Ministry of Culture, Sports and Tourism to revise the Medical Service Act and Tourism Promotion Act. The Korean medical tourism industry has focused on cosmetic surgery and cancer cure. The Korean government limited the proportion of foreign inpatients to 5% in tertiary hospital and to 8% in general hospital to ensure short waiting time for Korean patients. In 2013, a convergence business model for medical tourism was proposed to maximize the profits (12). KHIDI forecasted that the treatment revenue from foreign patients would increase 42.0% on an annual average and the non-treatment revenue would be capable of reaching 980.2 billion won at most with additional revenues produced by the convergence business model, as shown in Table 1.

**Table 1:** Revenue scenarios of medical tourism based on a convergence business model Unit: 100 million won

***Variable***		***2012***	***2013***	***2014***	***2015***	***2016***	***2017***	***Total***
Treatment revenue (①)		2,684	3,696	5,236	7,428	10,552	15,010	41,992
Non-treatment revenue (②)	Natural increase	1,934	2,478	3,191	4,110	5,292	6,815	21,886
	Scenario A		2,610	6,261	4,765	6,418	8,627	25,951
	Scenario B		2,698	3,757	5,202	7,168	9,802	28,628
Total revenue from medical tourism (①+②)	Natural increase	4,618	6,175	8,428	11,537	15,844	21,825	63,808
	Scenario A		6,307	8,767	12,193	16,969	23,637	67,873
	Scenario B		6,394	8,993	12,630	17,720	24,812	70,550

Notes: Scenario A refers to the stage of completion of a standard business model appropriate to treatment moving route by patient types and its application. Scenario B refers to the promotion stage of the business model to diversify programs by patient type and manage specialized consortium, center and complex by model. The total revenue from medical tourism is the sum of the treatment revenue and non-treatment revenue by scenario.

Source: Korea Health Industry Development Institute, 2013.10.31

Furthermore, efforts were made to create Jeju as the focal point of Asian medical tourism by establishing the Greenland International Hospital, which is a medical complex providing health care, relaxation and cutting-edge medical research and development services, to strategically regulate medical treatment. However, the delayed enforcement of the 2016 Framework Act on Service Industry Development re-affirmed conflicting views on the privatization of medical industry emerging from the convergence of health care and tourism. The government withdrew the approval in April 2018 due to the possibility of infringing on the nature of public health despite those policies emphasizing the privatization of medical system. This approach adequately represents the importance of policy for the medical tourism industry in a country. In 2016, the Korean government established the Comprehensive Plan for the Attraction of Foreign Patients and Overseas Expansion of Medical Care and the 5-year plan on Medical *Hanryu*. The major policies to promote medical tourism are shown in Table 2.

**Table 2:** Major policies for the promotion of medical tourism in Korea

***Year***	***Policy***	***Contents***	***Authority***
2000	Strategic vision and development for health care industry	Implementation of health tourism business in Korea - Construction of a website to advertise medical institutions	Ministry of Health and Welfare
2001		Designation of facilities for medical tourism - Kyunghee University, Jaseng/Conmaeul/Kyungjoo Oriental Hospital	Ministry of Health and Welfare
2004	Selection of 50 projects for the development of health care industry	Selection of projects for the revitalization of high value-added medical tourism industry	Ministry of Health and Welfare
2006	Medical industry advancement strategy report	Approval of for-profit business by medical corporations and suggestions for the inclusion of health care among service industries	The Presidential Commission on Healthcare Industry Innovation
2007	Participation in medical tourism industry	Initiation of the Korea International Medical Service Conference	Korea Tourism Organization, Korea Health Industry Development Institute
2008	Review of the introduction of for-profit medical corporations	Preparation of revitalization plans to attract foreign patients	Ministry of Economy and Finance
2009	Designation of global health care as a new growth engine	Preparation of infrastructure for the overseas expansion of hospitals and attraction of foreign patients	Ministry of Health and Welfare
	Revision of the Medical Service Act	Approval to attract foreign patients and encourage placement activity,	Ministry of Health and Welfare
	Revision of the Tourism Promotion Act	Distribution of funds for advancement of medical tourism and provide reasons for additional assistance	Ministry of Culture and Tourism
2011	Initiation of the tourism industry as a new growth engine	Implementation of overseas marketing and diversification programs for medical tourism products	Ministry of Culture and Tourism
2013	Revision of enforcement of the Tourism Promotion Act	Approval for the management of medical hotels by medical corporations, suggestions for a convergence business model	Ministry of Culture and Tourism
2015	300,000 foreign patients	Improvement of medical safety in Korea, enhanced market transparency to attract foreign patients, advanced service quality and increase patients’ convenience	Ministry of Health and Welfare
2016	The Framework Act on Service Industry Development	Installation of the Committee on Overall Policy for Service Industry for health care regulation, Withdrawal of regulations for active investment and job creation, implementation of the Comprehensive Plan for the Attraction of Foreign Patients and Overseas Expansion of Medical Care, and the 5-year plan on Medical Hanryu and 5 major tasks, enforcement of the Act on International Medical Business Assistance	Mainly controlled by Ministry of Health and Welfare

Notes: The analysis of authors is added to the source.

Source: Seungah Cho, Seungdam Choi (2016), A Preliminary Study on Problems of Revitalization Policy on Medical Tourism and Response: Drawing Implications and Analysis Foreign Cases. *Journal of Tourism Sciences*, Volume 48, 7, pp.165∼181. [In Korean]

According to the Korea Health Industry Development Institute, the economic value of attraction of foreign patients, based on policies for the medical tourism industry, “the revenue of medical treatment derived from foreign patients will reach 1.5 trillion won in 2017 and the accumulated revenue from 2013 to 2017 will be around 4.2 trillion won as it increases 42.0% on annual average.” Table 3 presents the medical treatment revenues derived from overseas patients, along with the total number of foreign patients and their nationality. Table 4 lists the average expenditure per patient according to nationality.

**Table 3:** Economic value and current status of foreign patients visiting Korea

***Variable***	***2009***	***2010***	***2011***	***2012***	***2013***	***2014***	***2015***	***2016***	***2017***
Total number of patients	60,201	81,789	122,297	159,464	211,218	266,501	296,889	364,189	321,574
Total medical treatment revenue	547	1,032	1,809	2,673	3,934	5,569	6,694	8,606	6,399
Total number of patients’ countries of origin	139	163	180	188	191	190	187	186	190

Source: Statistical analysis of foreign patients in 2017, Korea Health Industry Development Institute, 2018

**Table 4:** Average expenditure per foreign patient by country

***Variable***	***1***	***2***	***3***	***4***	***5***	***6***	***7***
Country	Kuwait	The United States	UAE	The Philippines	China	Vietnam	Thailand
Expenditure	43,740,244	27,568,596	23,352,807	9,522,257	9,403,639	6,967,933	6,528,594

Source: A 2017 survey of the role of foreigners in Korean medical wellness, Korea Tourism Organization, 2017

As shown in the Tables 3 and 4, the Korean medical market failed to increase in terms of the number of foreign patients and medical treatment revenues in 2017 due to several issues, such as the political and economic barriers between Korea and China in which the Chinese government prohibited visiting to Korea for economic retaliation (13). The average expenditure per foreign patient in Kuwait, the U.S., and UAE was ranked at the top followed by the Philippines and China. The expenditure excluding medical interventions was mostly attributed to cultural experience, shopping and leisure.

Policies for the Korean medical tourism industry can be divided into three eras: discussion of the medical tourism industry policy (2005∼2008); revitalization measures and amendment of the Medical Service Act and Tourism Promotion Act to foster the medical tourism industry (2009∼2013); and attraction of 800,000 foreign patients and implementation of industry development strategy and plans to attract foreign patients and overseas expansion of health care (2014∼present). It is noteworthy to soften regulations to address medical services and tourism through a transition period. The changes in the implementation of regulations are listed in Table 5.

**Table 5:** Changes in the regulations for the revitalization of medical tourism industry

	***License***	***Inpatient admission rate***	***Insurance company participation in patient attraction***	***For-profit corporation***	***Remote treatment***	***For-profit business***	***Medical advertisement***
Status	Only domestic	Tertiary 5%; General 8%	Prohibited	Non-profit corporation	Not allowed	Restrictions on for-profit business by medical corporations	Foreign language advertisements prohibited
Revision discussion	Hiring required under certain standards	Proposal to relax governmental regulations	Withdrawal of the Act on International Medical Business Assistance	Reinforcement of competitiveness	Passage of the revision of Medical Service Act by the cabinet	Revision of incidental business implementation and partial approval of for-profit corporation	Foreign language advertisements allowed in five areas

Source: Establishment Plan on the 2030 Future Development Strategy for Realization of Smart Daegu City

### Status of Korean Medical Institutions Attracting Foreign Patients

A number of patients visit foreign countries for medical service. Among foreign patients visiting Korea in 2014, the number of patients from China (29.8%) ranked first, followed by the U.S. (13.3%), Russia (11.9%), Japan (5.4%) and Mongolia (4.8%). The Korean government strengthened the infrastructure to attract foreign patients via legal and regulatory reforms and policy revisions in 2015, as shown in Table 3, to improve the market transparency and attract foreign patients (14). As a result, the number of foreign patients in 2017 was increased by 11.7% year-on-year. While the number of patients from Thailand and Japan increased, those from China, Uzbekistan and Kazakhstan showed a decline.

The types of medical facilities (the number of establishments/the number of registrations) established in Korea to attract foreign patients include tertiary hospitals (43/41), general hospitals (301/117), hospitals (1,466/226), dental hospitals (231/52), dental clinics (17,376/202), oriental medicine hospitals (312/30), oriental medicine clinics (14,111/151), clinics (30,938/839) and others (1529/6). The attraction and registration rate of foreign patients from tertiary hospital ranked the highest, followed by general hospital, dental hospital, hospital, oriental medicine hospital, clinic, dental clinic, oriental medicine clinic, and others, which suggested that many patients needed advanced medical services. As shown in Table 6, Korea offered professional medical tourism services to patients by race, disease and treatment. It is quite important to emphasize the merits of wellness tourism in Korea utilizing the concept of bio-health care based on genetics and stem cells (15). Accordingly, it is essential to promote policies and marketing to show that custom cutting-edge medical services are available in Korea.

**Table 6:** Annual average growth rate of foreign patients according to the type of medical facilities (2014∼2018)

***Variable***	***Dental hospital***	***Clinic***	***General hospital***	***Oriental medicine clinic***	***Oriental medicine hospital***	***Dental clinic***	***Hospital***	***Tertiary hospital***	***Other***
Growth Rate	40.1	33.8	28.0	25.8	23.4	20.7	19.6	15.2	15.2

Source: Statistical analysis reporting foreign patients in 2017, Korea Health Industry Development Institute, 2018

### Analysis on Foreign Patients

#### Major Treatment Categories

Analysis on the nationalities of foreign patients visiting Korea under major treatment categories is presented in Table 7. China ranked first among all treatment categories except in oriental medicine. Russia, showing a sharp increase, competed with the U.S. for the second place. Mongolia and Kazakhstan ranked fourth and fifth, respectively. It is remarkable that Japan showed a relatively low participation rate in all categories except plastic surgery, dermatology and oriental medicine. It is supposed that cultural context and racial similarities play a significant role in determining patients’ destination for medical treatment (16). The significance of various factors determining the medical service type depends on the nationality and culture rather than convenience or ease of access (17). This analysis provides a detailed empirical basis to facilitate the choice of medical category for further consideration in future policy-making decisions.

**Table 7:** Top five nationalities under major treatment categories

***Medical Subjects***	***1^st^ rank***	***2^nd^ rank***	***3^rd^ rank***	***4^th^ rank***	***5^th^ rank***
Integrated					
Internal medicine	China	The United States	–	Kazakhstan	Mongolia
Plastic surgery	China	Japan	Thailand	The United States	Viet Nam
Dermatology	China	Japan	The United States	Russia	Kazakhstan
Medical check-up	China	Russia	The United States	Kazakhstan	Mongolia
Orthopedics	China	The United States	Russia	Mongolia	Kazakhstan
Oriental medicine	Japan	China	The United States	Russia	Kazakhstan
Obstetrics and gynecology	China	The United States	Russia	Mongolia	Kazakhstan
Dentistry	China	The United States	Russia	Japan	Viet Nam
Neurosurgery	China	Russia	The United States	Kazakhstan	Mongolia
Ophthalmology	China	The United States	Russia	Kazakhstan	Mongolia
General surgery	China	Russia	The United States	Kazakhstan	Mongolia
Pediatrics	China	The United States	Russia	Mongolia	Kazakhstan
Otorhinolaryngology	China	The United States	Russia	Kazakhstan	Mongolia

Source: Statistical analysis of foreign patients in 2017, Korea Health Industry Development Institute, 2018

### Satisfaction Levels in Korean Medical Tourism

For 10 years, tourism resources in Korea were abundant and the quality of medical facilities and services has improved. It seems that this trend raised the satisfaction level of foreign patients. According to the 2018 Foreign Patient Satisfaction Level Survey with 1,200 foreign patients, conducted by Whosaeng Newpapers, medical technology ranked the highest with 90.5 points and perceived satisfaction level about the overall medical service was 90.2 points on average (18). This result is consistent with the medical tourism arena in Korea, and the result of a survey about overall satisfaction levels in this country, in which the satisfaction level of the patients was asked, and accordingly the satisfaction was high for patients coming from different countries, Russia showed the most positive results followed by China, the U.S. and Japan (19).

In the selection of medical tourism, foreign patients considered medical technology (41.5%), reputation (18.4%), language service (13.0%) and medical equipment (11.0%). Medical tourism prioritizes medical treatment and regards other experiences and wellness tourism such as thermal springs, spa and leisure activities as additional features. Accordingly, the investment in marketing for patients from developed countries can produce long-term profits (20). A strategic education program is required to accurately inform the merits and risks of medical tourism (21). For instance, the Medical Korea Information Center was launched in December 2018 at Incheon airport to provide professional services to overseas patients. Foreign patients can make reservations for hospital and obtain the information about medical services they want, hospital location, treatment cost, and attraction points and accommodation after professional medical consultation through the kiosk. Moreover, they can have experiences of finding Korean traditional teas suitable to them and testing their skin type and healthiness (22). This effort is a representative example of active marketing interventions.

### Issues

Although the reformation of policies and strategies in medical tourism has been fulfilled by domestic advertisements in only five restricted areas in terms of the promotion of policies, coherent promotional efforts at the national level are needed (23) (Medical advertisements are prohibited in Korea except 5 areas, the market only for foreigners, bonded area, international airport, trade port, and duty free shop in Jeju island, according to the Medical Service Act, article 56). In medical treatment, the regulation of regenerative medicine via stem-cell therapy should be revised. In terms of tourism, as more patients visit from Middle East and Southeast Asia, an in-depth understanding of cultural taboos about surgery and food is imperative (24). Comprehensive discussions are needed to develop a new vision to promote the Korean medical tourism industry. Finally, the communication challenges arising from the lack of coordinators and translators equipped with the knowledge on medical service hinder the capabilities of medical tourism sector in South Korea.

## Conclusion

This study investigated the current policies and the status of medical tourism industry in South Korea. According to the collected information, it is likely that Korea has invested a considerable amount of money and has so far spent remarkable time and effort for the development of medical tourism sector. However, there is still need for more strategies and practices in this arena which are suggested here as managerial implications.

The medical tourism industry began in Korea in 2009. The Korean government has established and revised policies for medical tourism, actively focusing on the attraction of foreign patients. The number of patients has increased continuously and now the Korean medical tourism industry aims to attract 800,000 foreign patients by 2020. While there have been improvements in the growth of the industry, the needs of patients from various countries and culture have been diversified and their standards on the quality of medical tourism have been increased, that roots in the cultural and family preferences (25, 26).

Accordingly, to make the Korean medical tourism industry be top-notch in a sustainable way, there is a necessity to revise policies, based on 10-year statistics, to meet diverse needs of foreign patients at the international level. Firstly, it is suggested to establish the medical tourism information system and brand the Korean medical tourism offering a joint treatment of western and oriental medicine. Secondly, it is recommended to prepare the expansion of the industry to the medical wellness tourism industry by training global healthcare experts (27). Thirdly, it is encouraged to organize tailored medical services considering various backgrounds and culture of foreign patients. Upon the improvement of policies and strategies following above suggestions, the Korean medical tourism industry would be more invigorated and have a chance to be a high value-added industry. These recommendations would work as a basis for the Korean medical tourism industry to develop and be top-grade at the global level.

## Ethical considerations

Ethical issues (including plagiarism, informed consent, misconduct, data fabrication and/or falsification, duplicate publication and/or submission, and redundancy) have been completely addressed by authors.
